# Cryo-electron tomography related radiation-damage parameters for individual-molecule 3D structure determination

**DOI:** 10.3389/fchem.2022.889203

**Published:** 2022-08-30

**Authors:** Han Xue, Meng Zhang, Jianfang Liu, Jianjun Wang, Gang Ren

**Affiliations:** ^1^ The Molecular Foundry, Lawrence Berkeley National Laboratory, Berkeley, CA, United States; ^2^ Beijing National Laboratory for Molecular Science, Institute of Chemistry, Chinese Academy of Sciences, Beijing, China

**Keywords:** radiation damage, cryo-electron tomography, single-molecule 3D density map, cryo-EM, protein structure, individual molecule structure

## Abstract

To understand the dynamic structure–function relationship of soft- and biomolecules, the determination of the three-dimensional (3D) structure of each individual molecule (nonaveraged structure) in its native state is sought-after. Cryo-electron tomography (cryo-ET) is a unique tool for imaging an individual object from a series of tilted views. However, due to radiation damage from the incident electron beam, the tolerable electron dose limits image contrast and the signal-to-noise ratio (SNR) of the data, preventing the 3D structure determination of individual molecules, especially at high-resolution. Although recently developed technologies and techniques, such as the direct electron detector, phase plate, and computational algorithms, can partially improve image contrast/SNR at the same electron dose, the high-resolution structure, such as tertiary structure of individual molecules, has not yet been resolved. Here, we review the cryo-electron microscopy (cryo-EM) and cryo-ET experimental parameters to discuss how these parameters affect the extent of radiation damage. This discussion can guide us in optimizing the experimental strategy to increase the imaging dose or improve image SNR without increasing the radiation damage. With a higher dose, a higher image contrast/SNR can be achieved, which is crucial for individual-molecule 3D structure. With 3D structures determined from an ensemble of individual molecules in different conformations, the molecular mechanism through their biochemical reactions, such as self-folding or synthesis, can be elucidated in a straightforward manner.

## Introduction

The ability of soft-/biomaterials to respond to environmental changes or stimuli is essential for their unique function (Ha and Loh, 2012; Cheng and Li, 2013; Wu et al., 2017; Patel et al., 2019). To understand how the function is regulated by environmental stimuli, the structure and conformational changes of the soft-/ biomaterials are often required. Cryo-electron microscopy (cryo-EM) single-particle averaging (SPA) resolves the 3D structure of macromolecules at atomic resolution in their near native state (Method of the Year 2015, 2016; Nakane et al., 2020). However, the determined structure is static and is near the ground state of energy, which is insufficient to reveal the intrinsic molecular flexibility and dynamics (Villarreal and Stewart, 2014), especially for macromolecules with large-scale and multidimensional freedom (Zhang and Ren, 2012) or conformational changes during biochemical reactions, such as folding. Under these conditions, a method to determine the 3D structure of individual molecules, rather than the average of the selected population of homogenous molecules, is sought-after for understanding their large-scale structural changes with a continuum of conformation. Cryo-electron tomography (cryo-ET) provides a unique tool to reconstruct the 3D structure of individual objects, such as bacteria, cell sections, or even individual molecules, from a series of images acquired at a set of tilt angles (Lucic et al., 2013; Doerr, 2017). However, the resolution of cryo-ET 3D reconstruction is limited by the low image contrast (or low SNR), which is mainly controlled by the limited electron dose that can be tolerated by the sample before causing the radiation damage (Chen et al., 2020; Hylton and Swulius, 2021). The resolution of cryo-ET reconstruction can be improved by subtomogram/subvolume averaging (Bartesaghi et al., 2008; Schur et al., 2013; Schur et al., 2016), in which the 3D volumes of individual molecules/particles are cropped (from a low-resolution 3D reconstruction obtained from a tomogram containing a large number of particles), selected (for a homogenous population of particles), aligned, and then averaged to reduce noise and improve the resolution. This approach has been used to solve the high-resolution structure of relatively rigid proteins. However, it is not adapted in 3D reconstruction of soft and plastic proteins, such as antibody (Zhang et al., 2015a), lipoproteins, and proteins in folding. Thus, an approach for high-resolution cryo-ET 3D reconstruction (without averaging) of an individual molecule/particle is still expedient for structural biologists.

The high-energy electron beam can destroy the intrinsic structure of molecules before high-contrast images are acquired (Glaeser, 1999; Henderson, 2004). Although technologies and techniques have been developed to improve the image contrast, such as direct electron detectors (Jin et al., 2008; Li et al., 2013a; Lucic et al., 2013), phase plate (Murata et al., 2010; Hall et al., 2011; Glaeser, 2013; Danev et al., 2014), and computational algorithms (Zhang et al., 2012a; Wu et al., 2018; Palovcak et al., 2020; Frangakis, 2021), high-resolution 3D structure determination is still challenging, especially for that of an individual molecule. Here, we review the electron radiation damage theory and cryo-EM–related experimental parameters for a better understanding of the mechanism behind the phenomena of radiation damage. Through the discussion of each experimental parameter, we can optimize the experimental strategy to achieve high-contrast and high-resolution cryo-ET data, a basis for individual-molecule 3D structure determination.

## Phenomena of radiation damage

**FIGURE 1:**
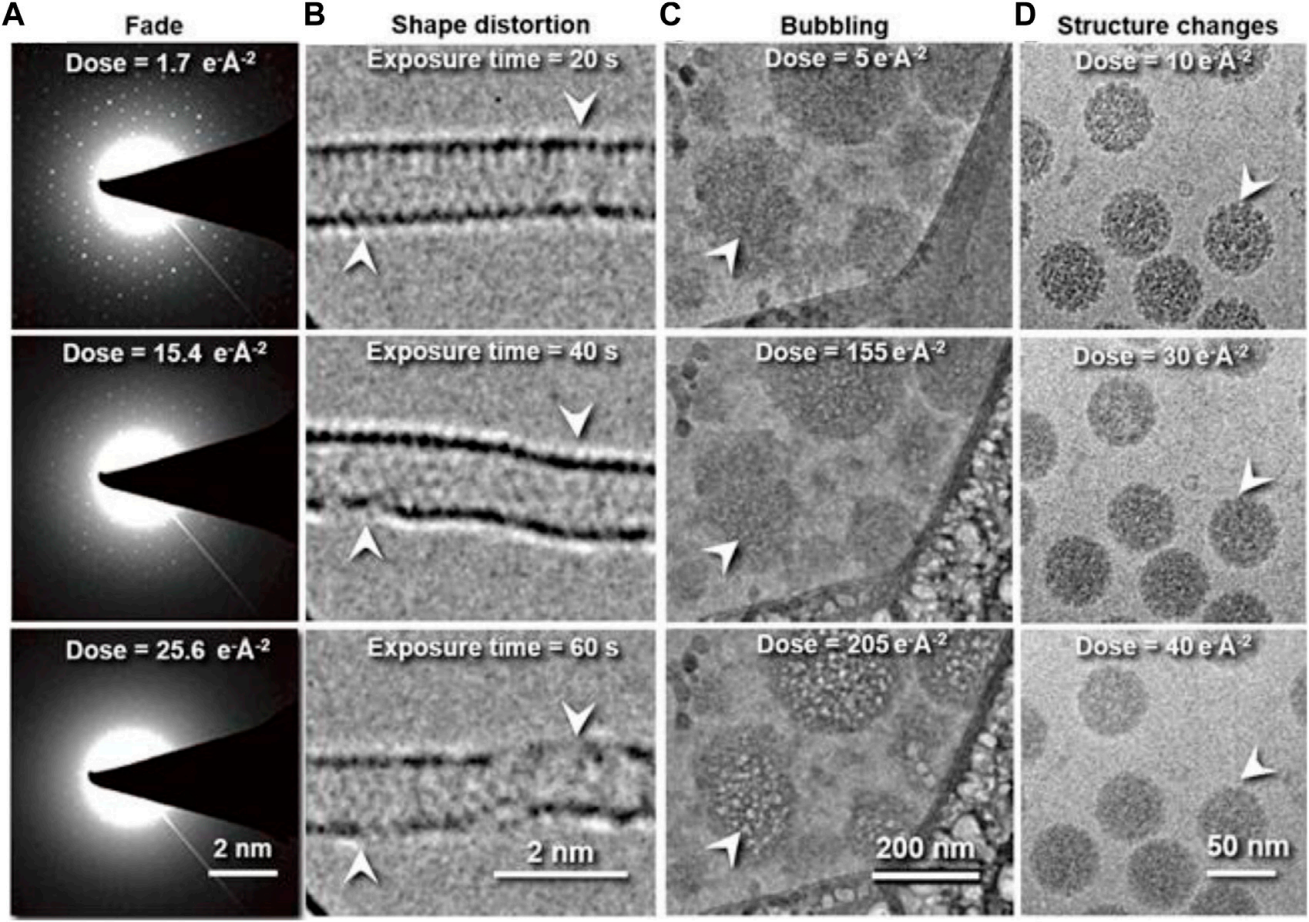
Typical phenomena induced by radiation damage under electron beam. **(A)** Reflection fade phenomena: Sequential electron diffraction patterns of purple membrane 2D crystals under a series of electron dose. As the dose increases, the spots fade, indicating cumulative radiation damage to the crystal. Reproduced with permission (Peet et al., 2019). **(B)** Distortion phenomena: Time series of SWNT images under constant electron beam irradiation. Reproduced with permission (Warner et al., 2009). **(C)** Bubbling phenomena: Bubbles appear with increased electron dose. Reproduced with permission (Daffner et al., 2020). **(D)** Structure-changing phenomena: Radiation damage in vitrified SV40 samples. Fine structural details are progressively lost in the SV40 particles as the electron dose increases from 10 to 40 e^−^ Å^−2^. Reproduced with permission (Baker et al., 1999).

In cryo-EM and cryo-ET data acquisition, the high-energy electron beams can change or even destroy the structure of soft-/biomacromolecules as a result of radiation damage (Hankamer et al., 2007; Baker and Rubinstein, 2010; Grant and Grigorieff, 2015; Hattne et al., 2018; Egerton, 2019). Radiation damage has been characterized by the following phenomena based on the type of samples. 1) Fading of diffraction spots in cryo-crystallography. This method is applied to electron diffraction of nanocrystals (MicroED) (Jones et al., 2018), thin-layer of protein 2D lattices (Taylor and Glaeser, 1974; Henderson et al., 1990; Ren et al., 2001), or proteins with helical symmetry (Sosa et al., 1997; Zhang et al., 2015b; Kellogg et al., 2016; Arragain et al., 2019; Egelman and Wang, 2021). For instance, the electron diffraction pattern of 2D catalase crystals gradually fades away with increasing electron doses (Figure 1A), indicating the cumulative radiation damage to the thin crystal (Peet et al., 2019). In this process, the radiation induces structural disorder, manifested first as the degradation of Bragg peaks at high resolution and a reduction in diffraction intensity, and finally the loss of the electron diffraction patterns. The failure to record high-resolution reflections prevents the structural determination of the crystals (Egerton et al., 2004; Clark et al., 2021). This method establishes the dose limit that induces radiation damage to the crystals. However, the criteria could overestimate the limit for individual molecules due to its sensitivity to the lattice order. 2) Shape distortion: For the low-dimensional crystal of macromolecules, such as graphene, carbon nanotubes, boron nitride, and molybdenum disulfide, consisting of low atomic number elements, radiation damage can be reflected in the change of the object shape in imaging (Chen et al., 2020). For instance, beam damage causes pristine single-walled carbon nanotubes (SWCNTs) to exhibit structural modifications, such as diameter changes, defect formations, inherent instability (shown in Figure 1B), and cumulated contamination (Warner et al., 2009). 3) Bubbling: For single-particle cryo-EM imaging, the radiation damage is more difficult to characterize than that of crystals. In this case, the changes in the surrounding environments are often used as a signal to predict the radiation damage (Hylton and Swulius, 2021). For example, microbubbles appear randomly in the sample Figure 1C (Daffner et al., 2020) when a hydrated biological specimen is under prolonged exposure to the electron beam. Similarly, bubbling is often observed in large biological particles (Cheng et al., 2014), such as virus particles (Wu et al., 2016; Wu et al., 2020). The gas bubbles have been identified as hydrogen gas by electron energy-loss spectroscopy. The gas is generated from radiolysis reactions under the electron beam (Leapman and Sun, 1995). When the growing bubbles reach the ice-vacuum interface, the hydrogen gas suddenly escapes from the vitreous ice, leaving behind an empty hole (Meents et al., 2010). Thus, bubbling is a sign of radiation damage (Glaeser, 2008). 4) Detailed structural change: The macromolecular shape is retained, but the molecular bonds are broken (Figure 1D) (Baker et al., 1999; Frank et al., 2002). These changes prevent the high-resolution 3D reconstruction of the true structure (Matthies et al., 2015; Hattne et al., 2018; Kato et al., 2021). For example, the structures of a pigment–protein complex determined by cryo-EM and X-ray free-electron lasers (XFEL) have shown inconsistent structural details. The cryo-EM structure showed a breakage of covalent bonds, progressive structural degradation, and the complete cleaving of the disulfide bond not found in the XFEL structure (Kato et al., 2021). The structural modification at this level is difficult to detect.

## Mechanism of radiation damage

**FIGURE 2:**
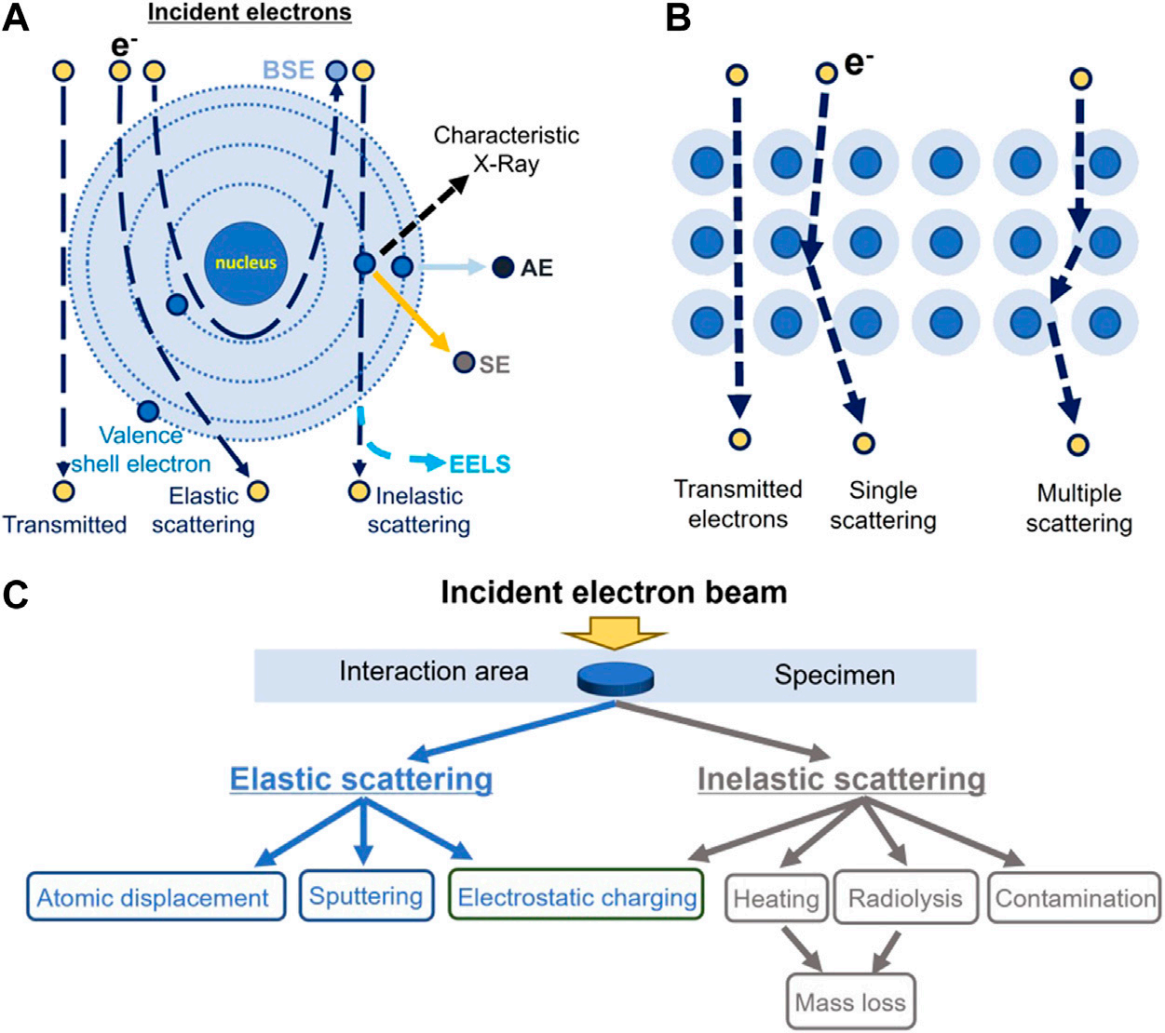
Schematic illustration of the interaction between the incident electrons and the atoms of specimen. **(A)** Schematic diagram of the described electron beam interacting with an atom in the sample, including the nucleus and electron cloud/shells. AE stands for Auger electron; BSE, for backscattered electron; SE, for secondary electron; and EELS, for electron energy-loss spectra. **(B)** Schematic diagram of the described scattering pathway of an incident electron within the sample. **(C)** Classification of radiation damage induced by electron beam.

In cryo-EM, the high-voltage electron beam traverses a thin layer of specimen and forms an image that carries the structural information of the specimen (Figure 2A). In this process, some incident electrons are transmitted directly through the specimen without any scattering (shown in Figure 2B) (Reimer, 1997). These electrons contribute to the background (white noise) of the image, which reduces the image contrast. Other incident electrons interact with the samples and are scattered. These scattered electrons play the most important role of generating the image contrast (Vainshtein et al., 2013), the higher the electron voltage, the smaller the scattering cross-section becomes (for both elastic and inelastic scattering) (Peng et al., 1996a; Peng et al., 1996b; Peet et al., 2019). The lower the percentage of scattered electrons (Meyer, 2014), the higher the percentage of incident electrons transmitted directly through the specimen without any scattering, resulting in a higher background intensity, which lowers the image contrast we get. On the other hand, the thicker the specimen, the higher the percentage of scattered electrons, and the higher the image contrast (provided that the electron beam can still traverse the specimen).

Scattered electrons carry more structural information (Peng et al., 1996a; Peng et al., 1996b; Ren et al., 1997; Colliex et al., 2006a) than X-ray or neutrons due to the fact that incident electrons are scattered by both the nucleus and its surrounding electrons in the sample (Spence, 2017). On the contrary, X-ray is only scattered by electrons, while neutron scattering is only contributed by the nucleus (Spence, 2017). Not surprisingly, electron scattering also induces more radiation damage to the sample with respect to X-ray or neutrons.

Based on the scattered electron energy, electrons can be classified as elastically or inelastically scattered electrons (Klein et al., 2012). The inelastically scattered electron is a major source of radiation damage (Williams and Carter, 2009) causing ionization, electron excitation, radiolysis, electrostatic charging, heating, mass loss, and contaminations (Figure 2C) (Egerton et al., 2004), which can also cause image blurring. Elastically scattered electrons can cause knock-out damage (Egerton et al., 2004). While traversing the sample, a few incident electrons can be scattered multiple times in a combination of elastic and inelastic scattering (multiple scattering as shown in Figure 2B). Electrons that have undergone multiple scattering cause a significant decrease in the image contrast, blurring the structural details of the sample image while inducing radiation damage (Korringa, 1994). For inelastic scattering, the incident electrons convert their kinetic energy partially into electronic excitations to the atoms of the materials (Cazaux, 1995; Jiang, 2016). The deposited energy can produce secondary-electrons for scanning electron microscope (SEM) imaging and excite X-rays for elemental analysis. The EELS can be used for element mapping (Glaeser, 1971; Egerton, 2013). Notably, as mentioned previously, the cross-sections of both elastic and inelastic scattering are increased by lowering the energy of the electron (operating voltage of the TEM), in which case a less percentage of electrons pass through the specimen without scattering and a higher percentage of the electrons contribute to the image. The experimental measurement on the bacteriorhodopsin and paraffin (C_44_H_90_) at liquid-nitrogen temperature showed that the elastic cross-section is increased by ∼201% and the radiation damage is increased by ∼157% at 100 keV in comparison to that at 300 keV (Peet et al., 2019). More details of the electron scattering theory can be found in the reference (Peng et al., 1996b; Peng et al., 1996c; Koster et al., 1997; Colliex et al., 2006b).

Upon depositing their energy to the sample, the electrons can damage the sample by changing or even destroying its structure. The damage from the destructive interaction between the incident electrons and the sample can be described in three stages: 1) Primary damage, in which the incident electrons ionize the sample, break its chemical bonds, and generate secondary electrons or free radicals; 2) secondary damage, in which the secondary electrons or free radicals migrate through the sample and cause chemical reactions; and 3) tertiary damage, in which the developed hydrogen gas within the sample causes large-scale morphological changes of the specimen (Baker and Rubinstein, 2010). The detailed discussion of the radiation damage from electron scattering is described in the following sections.

### Ionization and radiolysis

Incident electrons can excite atoms by depositing their energy and charges to the sample (Jiang, 2016). The deposited energy allows the rearrangement of the inter- or intramolecular bonds, resulting in changes of the molecular structure or the formed crystal structure by radiolysis (Grubb, 1974; Egerton, 2021). The study of the tobacco mosaic virus showed an example of the modification of covalent bonds in macromolecules by radiolysis (Fromm et al., 2015). Radiolysis initiates the decarboxylation process by breaking the hydrogen–oxygen bond and carbon–carbon bond in carboxylate residues. The breaking of the carboxylate residue damages nearby side chains, which can further change the secondary structure and flexibility of the molecule (Fromm et al., 2015).

**FIGURE 3:**
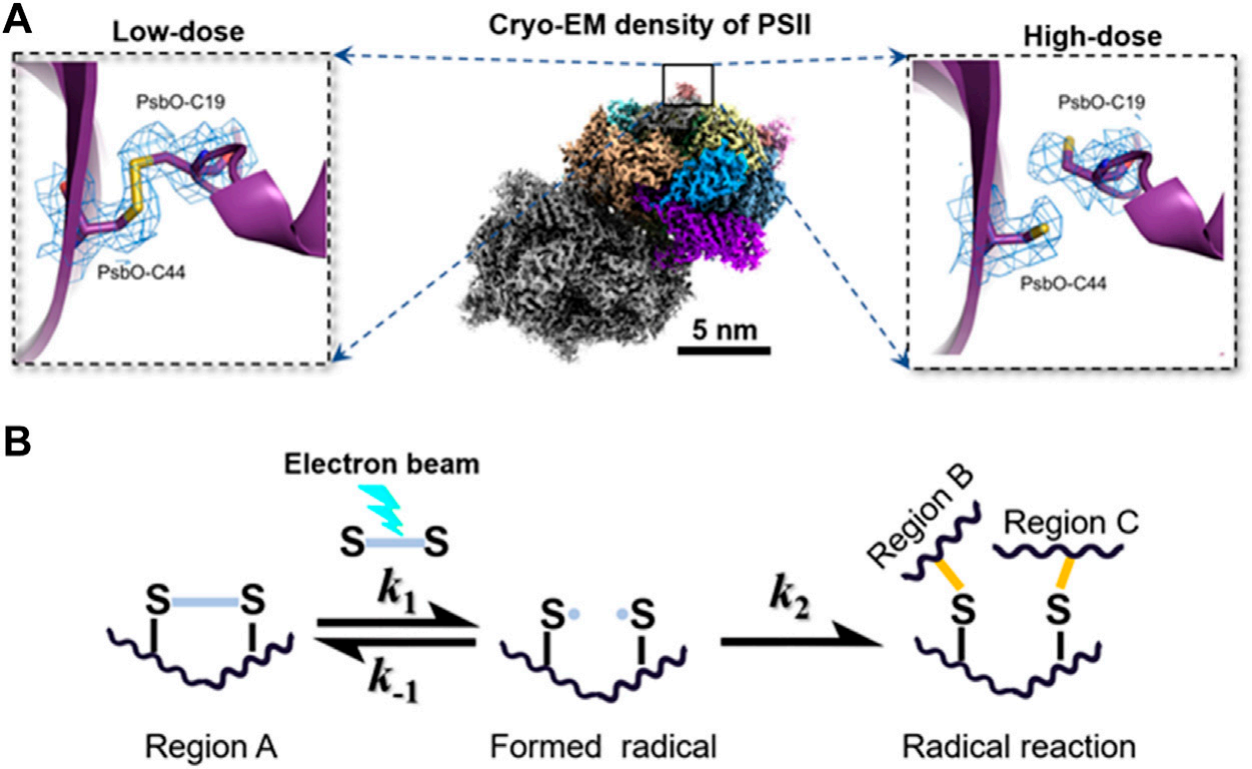
Electron beam–induced structural damages and radical reaction. **(A)** Broken disulfide bond in PsbO of PII at high-dose (left) and the disulfide bond recovered in PsbO of PII at the low-dose from single-particle averaging cryo-EM 3D reconstruction. Reproduced with permission (Kato et al., 2021). **(B)** Hypothetical scheme for radical reaction in the radiolysis damage. A radical reaction is initiated when an active site of disulfide bond is converted into radicals under the electron beam. The formation of radicals is a reversible reaction, in which the radicals can react with each other to recover the original biomolecule with a reaction rate constant of *k*_-1._ Increasing *k*_-1_ can inhibit the radiation damage effectively. However, if the radical in region A reacts with different radicals in other regions, the formation of a new reaction product is generally irreversible. The permanent damage in the biomolecule usually misleads the subsequent 3D reconstruction and reduces the structural resolution. Reproduced with permission (Kato et al., 2021).

The degree of radiation damage by radiolysis has three levels. 1) Local damage: the incident electron directly excites the local electrons of an atom, breaking its covalent bonds (shown in Figure 3A) (Kato et al., 2021). The damage leads to the appearance of free radicals in biological specimens (Mishyna et al., 2017). 2) Nearby damage: some free radicals can trigger a cascade of chemical reactions in nearby atoms (Grant and Grigorieff, 2015), and then transfer the free radicals to nearby atoms (shown in Figure 3B). 3) Structure crash: as energy and free radicals spread to nearby atoms through chain reactions, the chain reaction can terminate by generating new bonds between neighboring atoms and permanently changing the structure. For example, when the water molecule is dissociated to form a hydrogen and hydroxyl radical (H_2_O ⇒H• + OH•), some free radicals are converted back into H_2_O, while other free radicals close to the protein molecule react with the hydrogen atoms in the protein molecule to form hydrogen gas and a new free radical (OH•+ R-H ⇒ RO + H_2_). The released hydrogen coalesces into gas bubbles, which permanently damage the organic specimen (Leapman and Sun, 1995; Hankamer et al., 2007).

### Electrostatic charging

The electron beam causes electrostatic charging on the surface of the specimen. Negative charges can be caused by the accumulation of deposited incident electrons (Cazaux, 1986), while positive charges can be caused by the emission of secondary electrons of the insulating vitreous specimen (Egerton, 2021). These accumulated charges can cause electrostatic lensing by changing the trajectory of the incident electron, blurring the image and even generating a reaction force that induces mechanical movement of the sample, which in turn bends and distorts the thin specimen (Vinothkumar and Henderson, 2016).

### Heating

Cryo-EM imaging often causes an increase in temperature of the specimen, which manifests as melting or boiling (Dubochet et al., 1988). Heating is caused by the electron beam during the process when its kinetic energy is partially converted into thermodynamic energy of the atoms in the specimen *via* electron-phonon scattering (one type of inelastic electron scattering) (Johnston-Peck et al., 2016). Two factors can affect the local temperature rise of the specimen, that is, the total thermal energy absorbed by the specimen and the thermal conductivity between the specimen and the specimen-supporting film (Voss et al., 2021).

### Mass loss

The mass of the specimen can be reduced by the incident electron beam (Aronova et al., 2010) *via* two types of processes, that is, knockout and sublimation. For knockout, the incident electron directly hits the atom and kicks it out from the specimen (Kelly, 2002; Egerton et al., 2004; Gu et al., 2017; Su et al., 2019; Chen et al., 2020). Sublimation can be classified into direct sublimation and indirect sublimation. In direct sublimation, the energy directly increases the temperature of small molecules transforming the molecules from their solid state to their gaseous state, and releases them from the specimen (Dubochet et al., 1988). For indirect sublimation, radiolysis breaks the chemical bonds inside the macromolecules and transforms the fragments into small molecules, which are then released from the specimen surface in the gas phase (Egerton, 1999). For instance, water molecules in ice can be directly heated to a gas phase *via* sublimation into the vacuum environment, thus reducing the thickness of the specimen. Alternatively, the hydrogen–oxygen bond of water molecules can be also broken by radiolysis, generating many chemical intermediates (e.g., hydrogen radical, hydroxyl radicals, and hydrated electrons). These chemical intermediates can react with nearby damaged molecules released from the original macromolecules and form smaller new molecules, which then sublimate from the specimen (Le Caër, 2011). Both processes reduce the mass of specimens.

### Contamination

In cryo-EM imaging, the incident electron beam often causes contamination on the specimen surface through the calcination of organic molecules, which degrades the images of the targeted molecules (Isabell et al., 1999). The cause of contamination is very complex. One theory suggests that the organic molecules on the specimen surface are polymerized by the electron beams (Egerton et al., 2004). These polymers may attract each other and form an aggregation, increasing the thickness of the local area as irradiation continues (Ashfaq et al., 2020). Another explanation could be that the newly formed small molecules from the abovementioned chemical intermediates induced by radiolysis are absorbed onto the cold specimens with an uneven distribution of surface charge and then aggregated in the illuminated area of the specimen (Dubochet et al., 1988).

## Experimental parameters related to radiation damage

Radiation damage to protein molecules has been investigated for decades (Glaeser, 1971; Cosslett, 1978; Glaeser and Taylor, 1978; Glaeser, 1979; Misra and Egerton, 1984; Downing and Glaeser, 1986; Dubochet et al., 1988). The radiation damage can be evaluated by the following three methods based on the type of specimen (Li and Egerton, 2004; Egerton, 2009; Kawahara et al., 2009). 1) Diffraction pattern: the measurement of fading diffraction spots of crystalline specimens is often used to estimate the extent of radiation damage (Li and Egerton, 2004). This measurement is very sensitive to atomic displacements associated with structural degradation/amorphization. 2) Spectroscopy: EELS provides a powerful tool to monitor the change in chemical structure by recording the fingerprint of each chemical composition (Pal et al., 2021). EELS has been used to evaluate radiolysis-related radiation damage on the inorganic samples and organic semiconductors, but is rarely used for soft-/ biomaterials due to dose limitation (Egerton, 2009). 3) Structural changes: bubbling caused by radiolysis or changes of the fine structure of the macromolecules can be used as a sign to roughly evaluate the damage (Massover, 2010). The exact degree of radiation damage is unknown. However, with the same degree of radiation damage, a higher electron dose will certainly contribute to a higher image contrast and a higher resolution of 3D reconstruction. Each experimental parameter related to radiation damage is discussed in the following sections.

### Electron dose

The total number of incident electrons illuminating the unit area of the specimen, defined as the dose, has long been believed to be the only experimental parameter that governs the degree of radiation damage of the sample (Frank, 2009; de la Mora et al., 2020). However, the measured dose limit keeps changing over time (Wang et al., 2021a). For example, in the 1970s, the measured dose was ∼1 e^−^ Å^−2^ for the 7 Å reconstruction of the purple membrane 2D crystals (Egerton, 2014). In the 1980s, 4.0 to 4.5 Å resolution reconstructions can be obtained using the same limited dose (∼1 e^−^ Å^−2^) on n-paraffin and purple membrane samples (Henderson and Glaeser, 1985). Subsequently, even higher resolution images (3.5 Å and 2.8 Å) were reported using a dose of 20 e^−^ Å^−2^ on the purple membrane sample (Henderson et al., 1986a; Baldwin et al., 1988). In the 1990s, the 3.5 Å image of bacteriorhodopsin 2D crystals was acquired using a dose of 10–15 e^−^ Å^−2^ (Henderson et al., 1990). 30–35 Å 3D reconstruction of the HSV-1 capsid was obtained using 5–10 e^−^ Å^−2^ (Conway et al., 1993). In the 2000s, ∼11.5 Å 3D structure of 70S ribosome was achieved with a dose of ∼20 e^−^ Å^−2^ (Gao et al., 2003; Rawat et al., 2003). ∼8 Å structures of bacteriophage P22 capsid and ε 15 bacteriophage were solved with a dose of ∼30 e^−^ Å^−2^ (Jiang et al., 2003; Chen et al., 2008a). In the 2010s, ∼3.2 Å resolution structure of β-galactosidase was determined by a total accumulated dose of 45 e^−^ Å^−2^ (Bartesaghi, et al, 2014). ∼4 Å resolution structure of Slo2.2 Na^+^-activated K^+^ channel and human transcription factor IIH (TFIIH) were solved with a total dose of 40 e^−^ Å^−2^ (Hite et al., 2015; Greber et al., 2017). In the 2020s, 1.7 Å resolution 3D structure of human membrane protein, the β3 GABA_A_ receptor homopentamer, was solved with a total dose of 13.2 e^−^ Å^−2^, while a 1.22-Å resolution reconstruction of mouse apoferritin was solved with a dose of 40 e^−^ Å^−2^ (Nakane et al., 2020). Maps of RNA replication complexes were determined up to 8.5 Å resolution by cryo-ET subtomogram averaging with a total dose of 180 e^−^ Å^−2^ (Unchwaniwala et al., 2020), and the atomic resolution structure of ferritin was achieved with a total dose of ∼50 e^−^ Å^−2^ (Yip et al., 2020). Recently, the full-length *Tetrahymena* ribozyme was resolved at a resolution of 3.1 Å with a dose of up to 75 e^−^ Å^−2^ (Su et al., 2021); the GagT8I and apoferritin were determined up to 5.0 Å and 2.8 Å with the corresponding total doses of 122 e^−^ Å^−2^ and 102 e^−^ Å^−2^, respectively (Ni et al., 2022). Furthermore, the 3.6 Å resolution dNTPase was resolved with a total dose of 120 e^−^ Å^−2^ by cryo-ET subtomogram averaging (Bouvette et al., 2021). A summary of selected cryo-EM publications that contains the achieved 3D map resolution and their total dose is listed in Table 1. A summary of earlier publications can be found in a published article (Kudryashev et al., 2012).

**TABLE 1:** Selected cryo-EM/cryo-ET 3D reconstructions with corresponding experimental parameters.

Year	Authors and publications	Specimen	Method	Dose (e^−^ Å^−2^)	Dose rate (e^−^ Å^−2^ s^−1^)	Resolution (Å)
1975	Henderson, R. et al. (Egerton, 2014)	Purple membrane	2D crystal	1	---	7
1985	Henderson, R. et al. (Henderson and Glaeser, 1985)	n-paraffin	2D crystal	∼1	---	4∼4.5
1985	Henderson, R. et al. (Henderson and Glaeser, 1985)	Purple membrane	2D crystal	∼1	---	4∼4.5
1990	Henderson, R. et al. (Henderson et al., 1990)	Bacteriorhodopsin	2D crystal	10∼15	---	3.5
2003	Gao, H. et al. (Gao et al., 2003)	*E. coli* 70S ribosome	Single-particle averaging	∼20	---	11.5
2010	Liu, H. et al. (Liu et al., 2010)	Human adenovirus	Single-particle averaging	∼20	---	3.6
2010	Wu, S. et al. (Wu et al., 2010)	Muscle actin-myosin	Electron tomography	420	---	35
2011	Ge, P. et al. (Ge and Zhou, 2011)	Tobacco mosaic virus	Single-particle averaging	25	---	3.3
2013	Liao, M. et al. (Liao et al., 2013)	TRPV1 ion channel	Single-particle averaging	21	---	3.4
2014	Bartesaghi, et al. (Bartesaghi, et al., 2014)	β-galactosidase	Single-particle averaging	∼45	3	∼3.2
2015	Hite, R. K. et al. (Hite et al., 2015)	SloK^+^ channel	Single-particle averaging	40	8	4.5
2015	DiMaio, F. et al. (DiMaio et al., 2015)	20S proteasome	Single-particle averaging	30	3	4.5
2015	Zhang, M. et al. (Zhang et al., 2015c)	CETP–liposome complex	Individual-molecule (nonaveraging)	120	---	35
2015	Bartesaghi, A. et al. (Bartesaghi et al., 2015)	β-galactosidase	Single-particle averaging	45	5.9	∼2.2
2015	Campbell, M. G. et al. (Campbell et al., 2015)	20S proteasome	Single-particle averaging	53	7	2.8
2016	Zubcevic, L. et al. (Zubcevic et al., 2016)	TRPV2 ion channel	Single-particle averaging	57	5.7	4.0
2016	Walls, A. C. et al. (Walls et al., 2016)	Coronavirus S trimer	Single-particle averaging	∼53	∼7	4.0
2016	Merk, A. et al. (Merk et al., 2016)	Isocitrate dehydrogenase	Single-particle averaging	60	5	3.8
2016	Merk, A. et al. (Merk et al., 2016)	Lactate dehydrogenase	Single-particle averaging	60	5	2.8
2016	Merk, A. et al. (Merk et al., 2016)	Glutamate dehydrogenase	Single-particle averaging	40	2.6	1.8
2016	Liu, Z. et al. (Liu et al., 2016)	Packed PCV2 virus	Single-particle averaging	25∼27	---	2.9
2016	Yu, Y. et al. (Yu et al., 2016)	VLDL particles	Individual-molecule (nonaveraging)	150	---	35
2017	Greber, B. J. et al. (Greber et al., 2017)	Transcription factor IIH	Single-particle averaging	40	4.6	4.4
2017	Ertel, K. J. et al. (Ertel et al., 2017)	FHV RNA	Subtomo averaging	150	---	3.6
2018	Bartesaghi, A. et al. (Bartesaghi et al., 2018)	β-galactosidase	Single-particle averaging	45	5.9	1.5
2018	Draper-Joyce, C. J. et al. (Draper-Joyce et al., 2018)	A_1_ receptor–G_i_ complex	Single-particle averaging	50	4	3.6
2019	Zhang, K. et al. (Zhang et al., 2019)	Cytotoxin assemblies	Single-particle averaging	42	7	3.2
2019	Röder, C. et al. (Roder et al., 2019)	PI3K-SH3	Single-particle averaging	∼26	∼0.4	3.4
2019	Lei, D. et al. (Lei et al., 2019)	IDL particles	Individual-molecule (nonaveraging)	90	---	60
2019	Fan, X. et al. (Fan et al., 2019b)	Streptavidin	Single-particle averaging	50	19.5	3.2
2020	Bücker, R. et al. (Bucker et al., 2020)	Granulovirus	Single-particle averaging	4.7	235	1.55
2020	Bücker, R. et al. (Bucker et al., 2020)	Lysozyme	Single-particle averaging	2.6	130	1.80
2020	Fäßler, F. et al. (Fassler et al., 2020)	Actin complex	Subtomo averaging	170	---	9
2020	Nakane, T. et al. (Nakane et al., 2020)	β3 GABA_A_R	Single-particle averaging	13.2	3.4	1.7
2020	Nakane, T. et al. (Nakane et al., 2020)	Apoferritin	Single-particle averaging	40	2.1	1.22
2020	Yip, K. M. et al. (Yip et al., 2020)	Apoferritin	Single-particle averaging	∼50	∼2.7	1.25
2020	Hamdi, F. et al. (Hamdi et al., 2020)	Apoferritin	Single-particle averaging	28	∼1	2.7
2020	Klein, S. et al. (Klein et al., 2020)	SARS-CoV-2	Subtomo averaging	123	---	33
2020	Unchwaniwala, N. et al. (Unchwaniwala et al., 2020)	RNA complexes	Subtomo averaging	180	--	8.5
2021	Bouvette, J. et al. (Bouvette et al., 2021)	dNTPase	Subtomo averaging	120	---	3.6
2021	Wang, S. J. et al. (Wang et al., 2021a)	Ferritin protein lattices	Individual-molecule (nonaveraging)	272	8	22.9
2021	Turnbaugh, C. et al. (Turnbaugh et al., 2021)	20S proteasome	Single-particle averaging	50	6.5	3.8
2021	Su, Z. et al. (Su et al., 2021)	*Tetrahymena* ribozyme	Single-particle averaging	75	15	3.1
2021	Schuller, A. P. et al. (Schuller et al., 2021)	Nuclear pore complex	Subtomo averaging	145	---	25
2022	Ni, T. et al. (Ni et al., 2022)	GagT8I	Subtomo averaging	122	3	5.0
2022	Ni, T. et al. (Ni et al., 2022)	Apoferritin	Subtomo averaging	102	4.2	2.8

Table 1 shows that 1) the dose limitation measured in recent years is nearly hundred times higher than that measured earlier and 2) the dose limitation measured by SPA is much higher than that measured by 2D crystals. One may ask whether the radiation damage measured on the same protein in different forms (SPA vs. 2D crystal) could be different. In crystals, the chemical bonds, such as ionic and hydrogen bonds, formed between the protein molecules should have no fundamental difference from that formed within a protein for its secondary structure. The radiation damage on the bonds should be the same regardless of whether it is between the protein molecules or within the protein molecules. However, the fact that the observed dose limit measured from the crystal structure is lower than that measured from SPA could be explained as follows. The radiation damage on the protein surface can be detected sensitively by the quality of diffraction spots of crystals but cannot be precisely detected by SPA. The SPA accuracy in classification and alignment is limited by the computer algorithms. The resulted resolution is the averaged resolution, with the surface of the protein usually at a lower resolution than the inside, which is the so-called anisotropic resolution (Aiyer et al., 2021). Under this condition, the SPA resolution is difficult to be linked precisely to the dose limit. Additionally, a higher dose is often used for high-resolution 3D reconstruction *via* multiple-frame imaging using the direct electron detector (DED). One should note that only the first few frames of each image are used for the final high-resolution reconstruction, while the total frame/dose is used only for the initial low-resolution refinement/reconstruction (Cheng et al., 2015). In other words, the dose used for image acquisition is not equal to the dose for high-resolution 3D reconstruction. The actual dose used for the reported resolution (corresponding level of radiation damage) cannot be well-tracked from the publications.

Despite the abovementioned details, the changing dose in different experiments at different times suggests that dose limitation is a very complex function with respect to the radiation damage. In addition to the measuring methods (such as 2D crystal and SPA) and sample types, many untracked experimental parameters (such as ice thickness and buffer) may also contribute to radiation damage.

### Dose rate effect

The question whether the dose-rate correlates with radiation damage has been debated for decades. The experimental results have been controversial in the fields of both cryo-EM and X-ray crystallography. The experiments that support the argument that dose-rate is unrelated to radiation damage are in the majority based on the measurement of the fading of crystal diffraction spots against the total electron dose under various dose-rates (Glaeser, 1971; Taylor and Glaeser, 1976; Baker et al., 2010). In these experiments, a longer exposure time under the same total dose, that is, a lower dose rate, did not benefit high-resolution imaging (Glaeser, 1979), in which the fading of the critical diffraction spots was only related to the cumulative electron dose (Jeng and Chiu, 1984). Due to the fact that the maximum dose was less than 10 e^−^ Å^−2^, it was predicted that the structure of an individual protein molecule would never be possible (Henderson, 1992). A radiation damage experiment by X-ray diffraction intensities showed a similar conclusion, that is, the dose rate did not affect radiation damage at the same total dose. In this experiment, the changes in diffracted intensities between the first image and the third image, which showed radiation damage at the macroscopic level, are proportional to the total dose, but independent of the X-ray dose rate at flux densities up to 10^15^ photons s^−1^ mm^−2^ (Sliz et al., 2003). As the underlying principles of radiation damage to protein molecules remain unclear (Dubochet et al., 1988; Glaeser, 2008), the conclusion that the radiation damage is only dependent on the total dose, rather than the dose-rate is still controversial (Chen et al., 2008b; Karuppasamy et al., 2011; Hattne et al., 2018). At a higher dose rate, a higher temperature will be generated. The amount of increase in temperature is dependent on the conductivity of the supporting film. A higher temperature means a higher level of thermal vibration of the atoms, weaker chemical bonds, and more difficulty to heal the broken chemical bonds, and because of this line of reasoning, radiation damage has been thought to correlate with the dose-rate (Ravelli et al., 2002; Hattne et al., 2018).

The experiments supporting the importance of dose-rate on radiation damage include the following. The dose-rate effects were investigated by cryo-EM by collecting several series of cryo-EM images under different dose-rate and acquisition times, but with a same cumulative dose (Chen et al., 2008b; Karuppasamy et al., 2011). For instance, an individual-particle averaging cryo-EM study of the tobacco mosaic virus (TMV) showed less radiation damage in the 3D reconstruction with data acquired at the lower dose-rate based on images acquired under a total dose of 15 e^−^ Å^−2^ at dose-rates of 1.5 and 15 e^−^ Å^−2^ s^−1^, respectively (Chen et al., 2008b). This result is consistent with another cryo-EM experiment with a higher dose-rate range of 5–50 e^−^ Å^−2^ s^−1^ (Karuppasamy et al., 2011), suggesting that the secondary radiolytic effects, that is, radical recombination, play a role in the dose-rate effects.

Fading of the critical spot was often observed in the diffraction experiments of crystals of catalase 2D crystals (Taylor and Glaeser, 1974; Glaeser and Taylor, 1978) and 3D microcrystals (Nannenga et al., 2018). The fading phenomenon has been attributed only to the total dose-only–related radiation damage process, and not the dose-rate. One may argue against this viewpoint by the following logic. In the total dose-only model, each electron must permanently damage the local structure of a molecule, and the destroyed local structure should not change the location of molecule, that is, the overall order of the crystal should remain as it is. The undamaged molecules or undamaged portion of the molecules should still contribute to the high-resolution diffraction spots based on their perfect locations because diffraction is defined by the global order rather than the local structural disorder. In other words, the area that has not received any electrons would still contribute to the high-resolution spots during the process. In this case, the high-resolution spot intensities should be weaker instead of disappearing faster than the low-resolution spots. Thus, the dose-only model cannot fully explain the experimental observation, in which the reduction of high-resolution spots SNR is faster than that of the low-resolution spots.

An alternative explanation to the observed fading phenomena can be that the radiation damage is related to both the total dose and the dose-rate, as explained in the following. The incident beam on the illuminated area generates a higher temperature in the crystal. The higher the dose rate, the higher the thermal vibration of the crystal, the easier the disruption of the higher order crystallinity, and the faster the fading of high-resolution spots. The faster fading observed for the high-resolution spots than the low-resolution spots gives a clue that the temperature increase is the first effect before molecular damage. If so, allowing some time for the illuminated area to cool by using a pulsed beam instead of a continuous beam would give a higher dose toleration. A pulsed beam experiment has shown that the radiation damage is dependent on the time between electron pulses (VandenBussche and Flannigan, 2019). The other experiments support the notion that the dose rate correlated with the radiation damage involves the inorganic materials (Jiang and Spence, 2012), organic soft-materials (Kuei et al., 2020), and cells (Gordon Steel et al., 1986; Matsuya et al., 2018), in which self-healing has been proposed for the recovery of the damaged structure under a damaging threshold. The thresholds related to the dose-rate were studied in inorganic materials, biomolecules, and tissues (VandenBussche and Flannigan, 2019). The dose rate–dependent damage of cerium dioxide was found by scanning transmission electron microscopy (Johnston-Peck et al., 2016). The study of eight lysozyme crystals at room temperature and cryotemperature (100 K) showed that the tolerated dose is dependent on the dose-rate in a positive linear relationship (Southworth-Davies et al., 2007). The study of the disulfide bond Cys6-Cys127 showed that the electron density of the bond is dependent on the dose rate (de la Mora et al., 2020).

Due to the abovementioned controversial results from different experiments, whether radiation damage depends on the dose rate in cryo-EM or whether ultrafast electron pulses for TEM imaging is useful to mitigate radiation damage in soft biological materials remains under debate. A summary of selected cryo-EM publications that contain the achieved 3D electron density map resolution and their total dose and/or dose-rate is listed in Table 1 for readers to contemplate.

### Protein types

**FIGURE 4:**
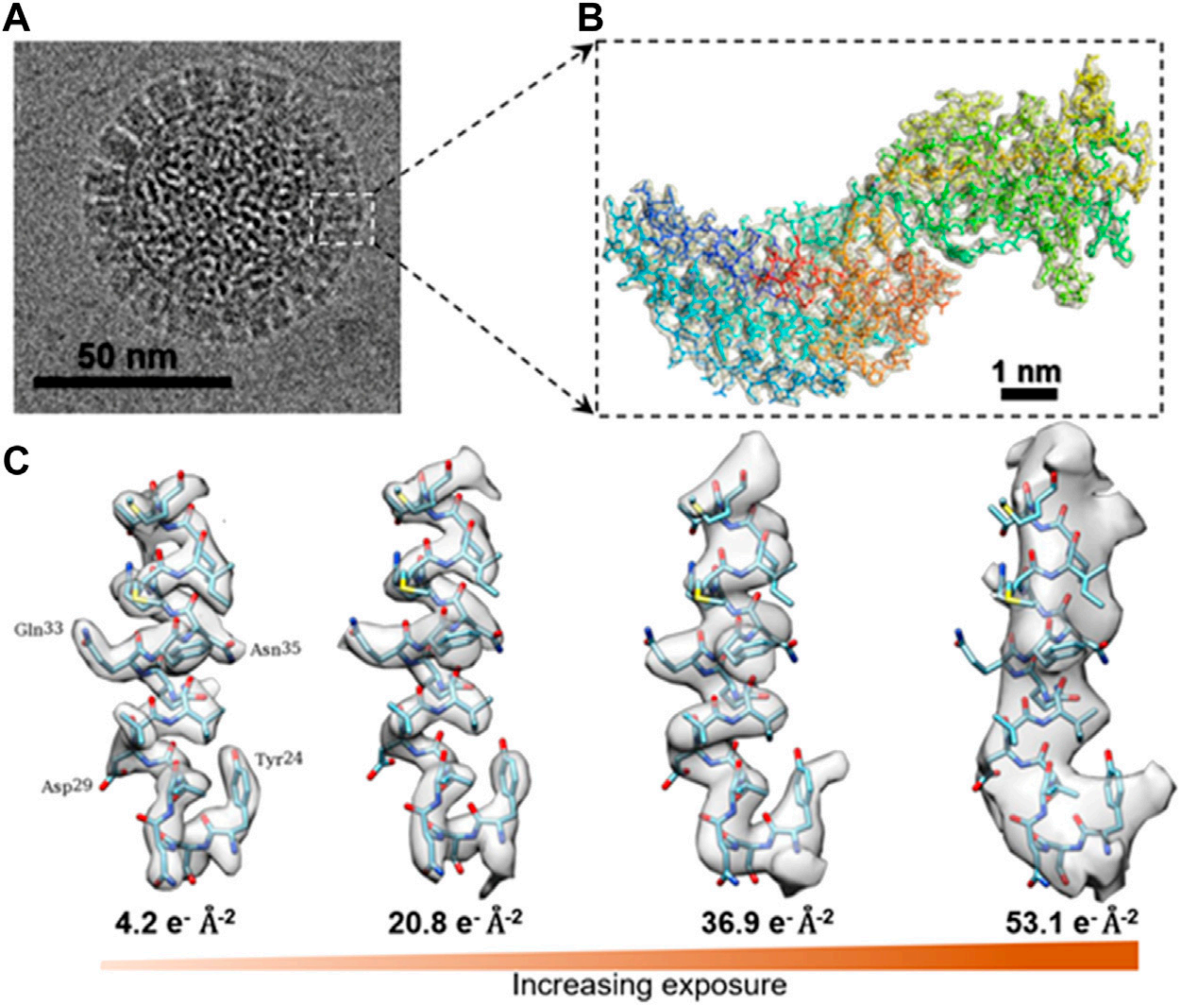
Cryo-EM 3D structure of rotavirus VP6 under different dose. **(A)** Aligned image of the rotavirus double-layered particle (DLP) imaged by cryo-EM. **(B)** Density of an isolated VP6 subunit is shown as a mesh along with the docked atomic model. The model is colored in blue for the N-terminus and in red for the C-terminus. **(C)** Surface rendering of an isolated small helix, in which the density of side chains fades with increasing exposure and dose deposited on the sample. Reproduced with permission (Grant and Grigorieff, 2015).

**FIGURE 5:**
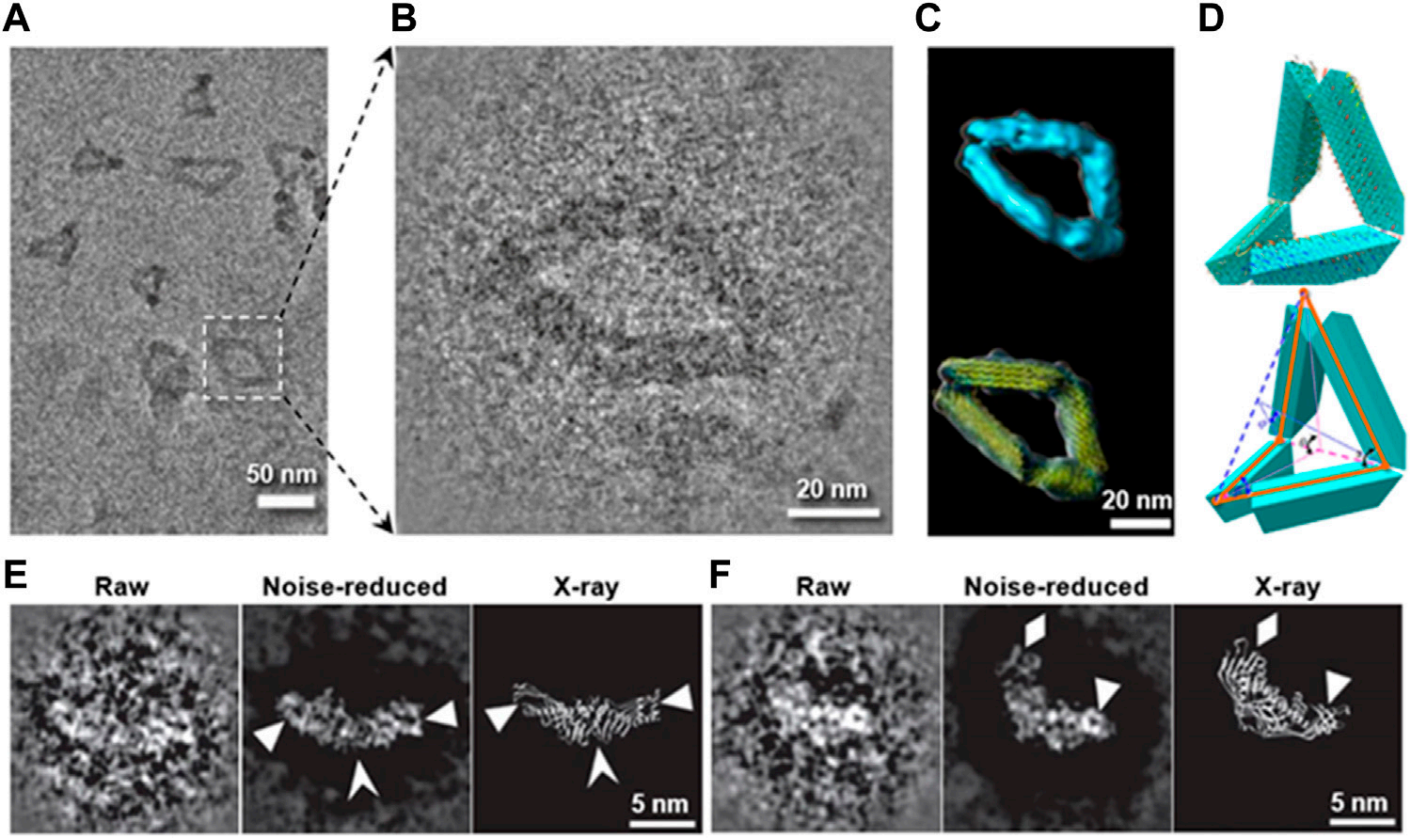
Preventing the radiation damage and raising the image contrast by heavy metal ion. **(A)** Overview cryo-positive-staining (cryo-PS) EM image of DNA origami in vitreous ice. **(B)** Magnified image of DNA origami boxed in **(A)**. **(C)** Final 3D reconstruction (up) and its model with flexible fitted structure (yellow ribbon). **(D)** Schematic diagrams of the defined internal angles within a particle. Reproduced with permission (Lei et al., 2018). **(E)** and **(F)** show two representative images of CETP imaged by cryo-PS (the left: a raw particle with reversed contrast, the middle: noise-reduced image of the raw particles, and the right: the X-ray crystal structure), in which the secondary structure details are indicated by arrowheads. Reproduced with permission (Zhang et al., 2012b).

In addition to the total dose and dose-rate as radiation damage–related parameters, one may ask whether the nature of biological specimens is related to the dose limit for radiation damage (Martynowycz et al., 2021); in other words, whether the intrinsic molecular properties, such as the molecular mass, diameter, density, stiffness, and local structure, affect the dose limit for radiation damage. The experiments that support the independence of radiation damage on the type of protein include the following. Radiation damage studies of protein crystals by cryo-EM reported that the dose limit is roughly the same for a very wide variety of organic and biological specimens (Henderson, 1990). In contrast, X-ray crystallography study suggests that there is no universally applicable dose limit for all types of protein crystals (Liebschner et al., 2015), which means that the dose limit depends on different protein crystals. The experiments that support the view that radiation damage depends on the type of protein or the molecule size or its physical environment (Kempner and Schlegel, 1979; Glaeser, 2016) include the following. The critical dose for the purple membrane at 7 Å resolution was ∼1.0 electrons Å^−2^ (Egerton, 2014), while 2D crystals of Connexin26 complexes were resolved at the resolution of 7 Å with an electron dose of 25 electrons Å^−2^ (Oshima et al., 2007). Moreover, in the SPA reconstruction, the dose for P22 bacteriophage capsid at 3 Å resolution was ∼37.5 e^−^ Å^−2^ (Hryc et al., 2017), while a helical reconstruction of TMV was revealed at 3.3 Å resolution, with a dose of 25 electrons e^−^ Å^−2^ (Ge and Zhou, 2011). One explanation that radiation damage may depend on protein types is that different proteins contain different percentages of negatively charged carboxylate residues, which are most susceptible to radiation damage. The radiolysis of carboxylate residues may lead to the deterioration of other intermediate and bulky side chains, as shown in Figure 4 (Grant and Grigorieff, 2015; Fromm et al., 2015; Bartesaghi, et al, 2014), resulting in a variety of radiation damage. As a matter of fact, to protect the charged carboxylate residues, a small amount of staining reagent, for example, uranyl formate, that can penetrate the molecular surface and bind to the carboxyl groups by the uranyl cation, may be introduced to reduce the radiation sensitivity, increase the tolerated dose, create more scattering from the heavy elements, and raise the image contrast of the organic soft-/biocompounds (shown in Figure 5) (Zhang et al., 2012b; Rames et al., 2014; Lei et al., 2018).

### TEM high-tension and defocus

Whether the dose limit is related to the operating voltage (high tension) of cryo-EM has been discussed before (Martynowycz et al., 2021). Some people believed that a higher voltage (300–400 kV) in cryo-EM can reduce the radiation damage with a higher dose limit (Schmid et al., 1992; Yalcin et al., 2006). The study of the electron elastic scattering cross-section from atoms and molecules in the 1.0 keV to 1.0 MeV energy range (Yalcin et al., 2006) showed that the higher the electron velocity (shorter incident electron wavelength) (Henderson and Unwin, 1975), the fewer interactions it has with the atoms of the specimen, and thereby a higher incident electron dose is tolerated for the same radiation damage.

The studies showed that the scattering cross-section decreases under higher tension (Peng et al., 1996b; Colliex et al., 2006a), resulting in decreased percentage of incident electrons being scattered; in other words, the signal containing structural information is decreased, especially for a thin sample. Increasing the incident electron beam will not contribute to the structural information, but rather to the background, which reduces the image contrast. Cryo-EM imaging at low-voltage (e.g., 30–80 kV) can increase the image contrast by increasing the scattering cross-section, in which a higher percentage of incident electrons contribute to the structural images, although the total tolerated incident dose is reduced. Moreover, low-voltage imaging reduces knockout damage (Egerton, 2014). However, due to the increased scattering cross-section, the percentage of inelastic scattering and multiple scattering are also increased. Under this condition, super-thin specimens (e.g., ∼30 nm), energy filters, and special cameras for low accelerating voltages become necessary for cryo-EM imaging.

Although the lower-voltage can increase the image contrast, this benefit may be canceled by the need to use a lower defocus value for imaging. The need arises because the lower the operating voltage (longer weave length) of the electron beam (Carter and Williams, 2016), the more oscillations there are in the contrast transfer function (CTF). As a result, 1) a higher percentage of structural factors are permanently eliminated (at the frequencies that CTF crosses zero) and 2) the envelope function drops faster at higher frequency, which means that the weight of the high-resolution information is reduced faster (the SNR of the high-resolution portion decreases). Under this condition, a lower defocus is required during cryo-EM imaging, which adversely contributes to the image contrast. Thus, the benefit from the lower voltage is limited.

### Temperature

The success in imaging the soft-/ biomolecules by cryo-EM is due to the low-temperature of the specimen. Based on thermodynamics, cooling the sample slows down the motion of the molecules, reducing the secondary damage of radical diffusion in radiolysis. Thus, the radiation damage is also reduced under the same radiation condition. Cooling the specimens to liquid nitrogen temperature (∼77 K) significantly limits the radiation damage from the electron beam (Glaeser, 2008). The question about whether an even lower temperature such as liquid helium temperature (∼4 K) would be better for preventing radiation damage has been studied a decade ago, and the results were controversial.

**FIGURE 6:**
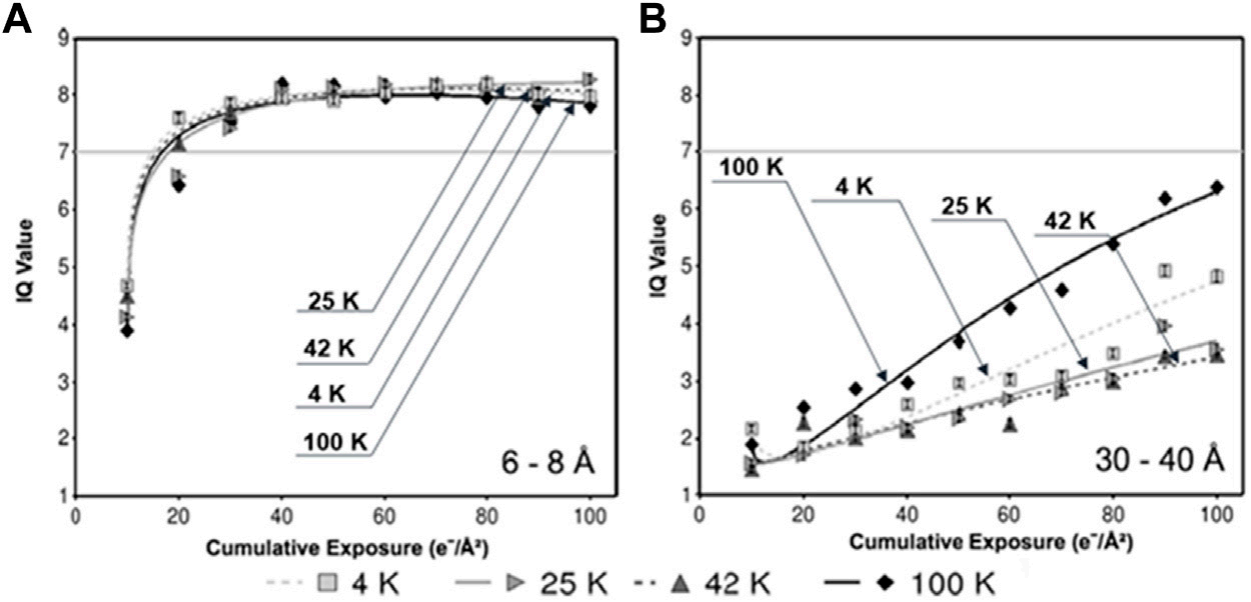
Radiation-induced decay of IQ values at different specimen temperatures. **(A)** IQ values within the resolution zone of 6–8 Å, **(B)** and zone of 30–40 Å, in which each data point represents the mean IQ value of all Bragg peaks within the specified resolution zone at the specified cumulative exposure at the specified temperature. Reproduced with permission (Bammes et al., 2010).

Experiments supporting an even lower temperature include the following. The study of the dose tolerance of *Caulobacter crescentus* cells at liquid helium temperature versus liquid nitrogen temperature showed that bubbling within the cell is slower at helium temperature (Comolli and Downing, 2005). Studies of 2D protein crystals showed that higher-order diffraction spots fade slower under near liquid helium temperature than under near liquid nitrogen temperature, suggesting the lower temperature further inhibits the radiation damage (Stark et al., 1996; Fujiyoshi, 1998; Naydenova et al., 2022). Cryo-ET study showed a modest improvement in preventing the radiation damage at intermediate temperatures (25 or 42 K) as shown in Figure 6 (Bammes et al., 2010).

However, a study by cryo-EM SPA reconstructions of heavy-chain apoferritin at near liquid nitrogen (85 K) and near liquid helium (17 K) temperatures showed no difference between these two temperatures (Pfeil-Gardiner et al., 2019), which is consistent with the cryo-EM study of the bacteriorhodopsin 2D crystals at liquid helium temperature (Stark et al., 1996). The result is also consistent with another cryo-EM study at medium resolution (3–5 nm) under the temperature of ∼12 and ∼82 K (Iancu et al., 2006). In this study, cryo-ET reconstructions of various biological samples (e.g., mesoplasma florum and liposomes) under a total dose between 10 and 350 e^−^ Å^−2^ showed worse results at ∼12 K than at ∼82 K (Iancu et al., 2006). Similarly, a study based on X-ray diffraction of biological samples showed no difference in the radiation damage at 50 K and at 5 K, although the damage can be reduced by a factor of ∼4 compared to 100 K (Meents et al., 2010).

The image contrast at near liquid helium temperature is lower than that at a relatively higher temperature (Iancu et al., 2006; Wright et al., 2006). The reason behind this phenomenon is unknown. An explanation could be the transformation of the amorphous ice at near liquid helium temperature into high-density amorphous (HAD) ice (Mishima et al., 1984)**,** resulting in a reduction of the difference between the ice density and the protein density, which gives rise to a lower image contrast. Moreover, the collapse of vitreous water at a higher density state (Wright et al., 2006) can exert mechanical stresses on the protein or crystal, resulting in increased beam-induced motion with liquid helium cooling (Wright et al., 2006; Pfeil-Gardiner et al., 2019).

### Substrate conductivity

Radiation damage induced by the accumulated charges on the specimen during imaging has been discussed in a published article (Jiang, 2016). After the incident electron beam leaves the specimens, charges created by the interaction may accumulate on the specimen surface (Jiang, 2016), especially for insulating specimens. When the accumulated charge reaches a steady state, image may be degraded by the electrostatic perturbation. Moreover, the accumulated charges produce local electric fields, and the electrostatic force may induce the release of mechanical strain, which results in the movement of the frozen specimen or destroying the thin layer of vitrified ice (Glaeser, 2008). Considering that the charge build-up may perturb the electron beam and cause specimen motion, pre-exposure was often used to reduce charging before imaging. Based on the abovementioned factors, a higher electrical conductivity of the substrate (vitreous ice or supporting film) should contribute to the prevention of radiation damage by eliminating the accumulated charges (Taylor and Glaeser, 1974). The conductivity of the specimen can be improved by imbedding conductive nanomaterials (Pantelic et al., 2012; Kleinerman et al., 2015), such as graphene nanosheets and carbon nanotubes in the sample, or using a supporting film with better conductivity than amorphous carbon supporting film, such as gold holey/lacey film (Russo and Passmore, 2014a) and graphene (Russo and Passmore, 2014b).

Additionally, the increased specimen/substrate electrical conductivity can also increase its thermal conductivity based on the Wiedemann–Franz law in physics. The higher the specimen conductivity, the less electrons are accumulated, and less energy is deposited in the local illuminated area. A higher electrical conductivity also leads to a smaller increase in the local temperature, with less thermal expansion and less beam-induced deformation and specimen drift/motion. Although, large-scale beam-induced deformations (Frank et al., 2002; Zheng et al., 2017) and specimen motion (Li et al., 2013a) can be partially corrected by computer algorithms, such as the “unbending” method in cryo-crystallography (Henderson et al., 1986b; Gil et al., 2006), the motion-correction methods based on the correlation of DED frames (Li et al., 2013a; Bai et al., 2013; Zheng et al., 2017; Zivanov et al., 2019), or the focused electron tomography reconstruction (FETR) method to reduce the deformation influence to 3D cryo-ET reconstruction by reducing the reconstruction image size (Zhang and Ren, 2012), minimal beam-induced deformation during data collection will still have superior image quality. Thus, a specimen with higher conductivity is still useful.

### Self-healing and sample recoverability

A study of DNA damages and repairs in human cells showed that the detected DNA damages are completely repairable under 4 MeV electron beam irradiation by using a high dose-rate with laser-generated ultrashort pulses (Babayan et al., 2017). One may ask whether the cryo-EM sample has a self-healing capability from radiation damage. The capability of self-healing from radiation damage have been reported based on the studies of atomistic simulation (Bai et al., 2010) on hard materials (Zhang et al., 2020) as well as soft-materials (Perepelkin et al., 2019). In the process of self-healing, the radiolysis-generated radicals react to recover the original molecule spontaneously (Phaniendra et al., 2015). Thus, the capability of self-healing should depend on the recovery time and the dose-rate. One experimental strategy to benefit from self-healing is to use a pulsed electron beam, which can give the sample a timespan for molecular self-healing. The potential benefits of using a pulsed electron beam to mitigate radiation damage has been speculated for decades.

By allowing a short time for specimen self-healing, the higher tolerated dose may further increase the cryo-EM image contrast. However, one should note that the usage of ultrahigh dose pulsed imaging does not mean that a higher dose-rate must be used, a lower dose-rate can also be used. The choice of dose-rate could be limited by other parameters, such as limitations of the DED, in which case the dose-rate cannot be too low for enough information to be collected for frame alignment or motion correction, or higher than the frame readout rate (Sun et al., 2021). Nevertheless, whether cryo-EM samples have self-healing or recoverability capability from radiation damage is still an open question. We hope this information can be helpful to readers to design their own strategy for minimizing radiation damage by taking advantage of the prospective self-healing properties of their samples.

### The thickness and size of vitreous ice

The physical size (thickness and diameter) of vitreous ice that spans across the holes in supporting carbon film should influence the radiation damage and dose limit (Zhang and Ren, 2012). However, its influence on the radiation damage has rarely been discussed. Two extreme cases can be used to understand why the physical dimension of the ice layer can influence the sample radiation damage. One extreme case is when the sample is imbedded in a bulk ice block, through which no incident electron can be transmitted. In this case, the bulk ice almost completely protects the protein from radiation damage. Although some electrons may carry the structural information of the proteins by elastic scattering from the sample, the transferred structure information is quickly destroyed by multiple scattering (Figure 2B), leaving no incident electrons for imaging. An opposite extreme case is when the ice layer is as thin as it is absent, in which case the electron beam interacts directly with the atoms of the sample. As a result, the sample can be easily damaged. In this case, all scattered electrons are 100% from the sample instead of partially from the sample and partially from the buffer/solution. In the normal case, when the sample is embedded in a thin layer of ice, the electrons scattered from the buffer/solution overlap with those from the sample, reducing the sample image contrast. Moreover, the thin sample also reduces multiscattering, which reduces the image blurring and increases the image sharpness.

**FIGURE 7:**
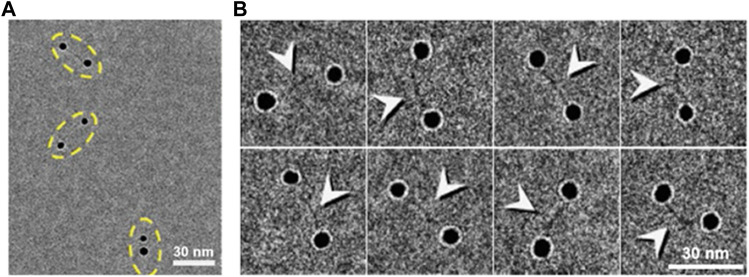
Cryo-EM images of dsDNA-nanogold conjugates. **(A)** Cryo-EM images of 5-nm nanogold particles conjugated to 84-bp dsDNA *via* a 50-thiol linker with thin vitreous ice. Pairs of nanogold were marked by yellow dashed ovals. **(B)** Eight representative cryo-EM images of the particles of DNA-nanogold conjugates. Polygonal-shaped areas are the nanogold particles were bridged by a fiber-shaped density (high-contrast densities were indicated by arrows), ∼20–30 nm in length and ∼2 nm in width. Reproduced with permission (Zhang et al., 2016).

**FIGURE 8:**
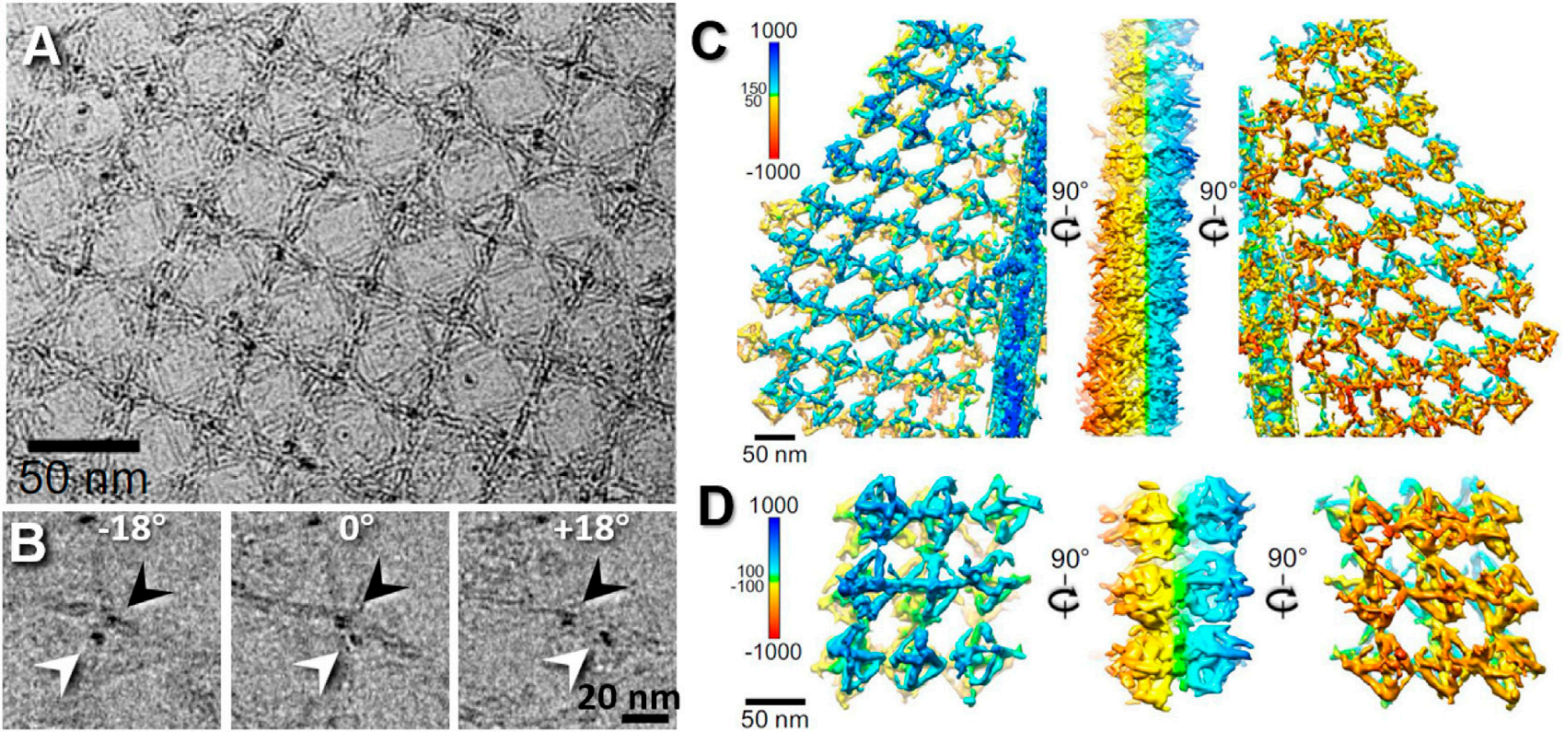
Missing-wedge–corrected 3D reconstruction of DNA origami double-layered (DL) lattice. **(A)** A cryo-ET image of a 2D DL lattice of DNA origami octahedral cage with ferritin protein imbedded in an extended sheet of vitreous ice. **(B)** Representative cryo-ET images showing the two ferritins in the top and bottom layers at tilted angles (black and white arrows) in a DL lattice. **(C)** Selected lattice area of cryo-ET 3D reconstruction (without averaging). **(D)** Representative 3D density maps after missing-wedge correction by LoTToR. Reproduced with permission (Wang et al., 2021b).

In cryo-EM, the ice thickness is usually, in the abovementioned two cases, mostly within a range of 20–500 nm, in which the radiation damage is in between the abovementioned two extremes (shown in Figure 7) (Zhang et al., 2016). In other words, the radiation damage should be a function of ice thickness. The thinner the ice, the higher the image contrast we get, but with less tolerance to radiation damage. In contrast, the thicker the ice, the lower the image contrast we get, but with higher tolerance to radiation damage as more energy is needed to raise the ice temperature and to damage the imbedded proteins. Thin ice can be achieved by using lacey carbon film–coated grids instead of quantifoil grids, because various-sized holes (up to hundreds of microns) in lacey carbon grids provide a perfect match to the thinnest liquid film (i.e., largest size) that can be formed based on the buffer surface tension. To produce an even thinner ice film, a small amount of detergent (>0.1%) can be added into the sample solution to reduce the liquid surface tension if the detergent is compatible with the sample. Similarly, the area of the ice film should also be another function of radiation damage due to the higher heat capacity of the larger ice as described in the previous publications (shown in Figure 8) (Zhang and Ren, 2012; Lei et al., 2018; Wang et al., 2021b). Large area thin ice film can be achieved by using the lacey carbon film–coated TEM grid (Zhang and Ren, 2012; Segrest et al., 2015).

### Other techniques to overcome the effects of radiation damage

In cryo-ET imaging, some other techniques also help in preventing radiation damage by reducing the total dose while achieving the same level of image contrast. The implementation of these techniques can change the experimental imaging strategy. One technique is using dark-field imaging, in which the nonscattered electrons are excluded, leaving an image formed solely from the scattered electrons (Chiu and Glaeser, 1975). The dark-field image contrast has been reported to be about four times higher than that from bright field imaging (Krivanek et al., 2010; Tsai et al., 2016) with the same total dose. However, due to the difficulty in designing a beam stopper and installing it in the center of the objective aperture to block the central unscattered beam without blocking the scattering beam, the application of this method is limited. The second technique is phase-plate imaging, in which the phase of the electron wave is modulated by a thin film placed in the beam pathway to improve the imaging contrast by slightly changing the phase (Danev and Nagayama, 2001; Fukuda et al., 2009; Murata et al., 2010; Fukuda and Nagayama, 2012; Danev et al., 2014; Egerton, 2019; Malac et al., 2021). Recently, a high-intensity laser beam has been used to replace the thin film to modulate the phase of the electron wave. This laser phase-plate can avoid the weakness of the electrostatic charging or contamination presented in the thin-film phase-plate (Schwartz et al., 2019).

The third technique is the DED, which can improve the detective quantum efficiency (DQE) for high-contrast imaging (Li et al., 2013b; Fromm et al., 2015). Moreover, the fast DED allows movies taken with dose fractionation for the correction of specimen drift/motion, which reduces influence on the image resolution. The last frames in DED movies can also provide additional low-resolution information after high-resolution information is degraded (Cheng et al., 2015). This feature can improve the final 3D resolution and provide a precise tool to set the dose limit for preventing radiation damage. In other words, increasing numbers of initial frames (thus increasing total dose) can be used to determine the dose when the resolution starts to deteriorate. With the total dose less than this critical dose, the actual radiation damage will be less than that which can be measured by any averaging method or tool because many parameters, such as the flexibility of the molecule, image blurring (caused by drift/motion, charge, inelastic scattering, beam coherence, and defocus measurement/correction), and the accuracy of the image alignment and image noise, can also reduce the 3D reconstruction resolution as radiation damage does. A reliable measured resolution should be lower than the resolution limited by radiation damage.

**FIGURE 9:**
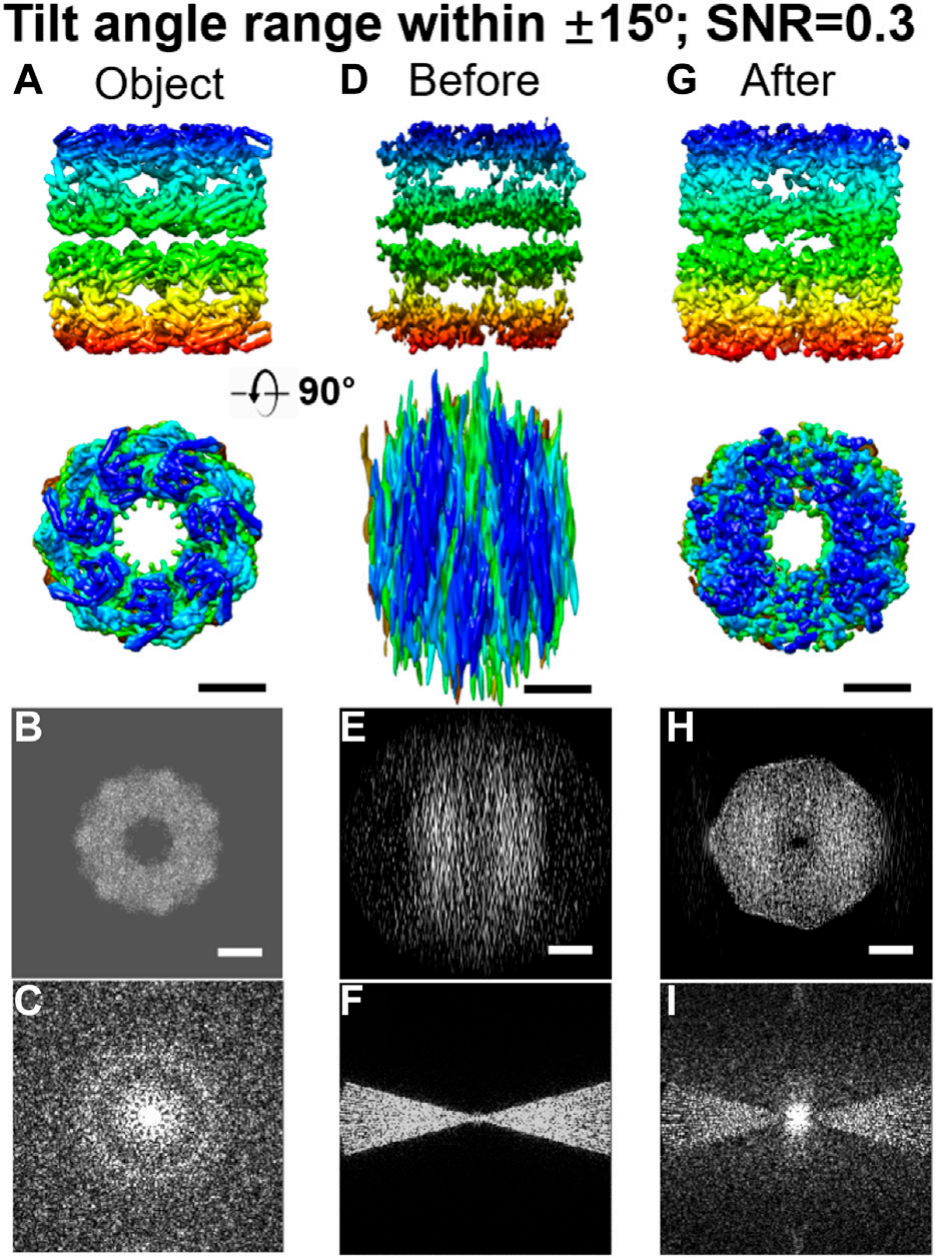
Missing-wedge correction on simulated 3D maps of reconstructed GroEL by LoTToR. **(A)** Two perpendicular views of the original object, a GroEL particle. **(B** and **C)** Projection and its Fourier transform of the 3D map along the X–Z plane. **(D)** Two perpendicular views of the initial 3D map, which was reconstructed from a noisy tilt series (SNR = 0.3) within a tilt angle range of ±15^o^ in steps of 1.5^o^. **(E** and **F)** Its corresponding projection and Fourier transform of the initial 3D map along the X–Z plane. **(G)** Final 3D map after missing-wedge correction (after 1,000 iterations), is shown from two perpendicular views. **(H** and **I)** Corresponding projection and Fourier transform of the final 3D map along the X–Z plane. Scale bars: 50 nm. Reproduced with permission (Zhai et al., 2020).

**FIGURE 10:**
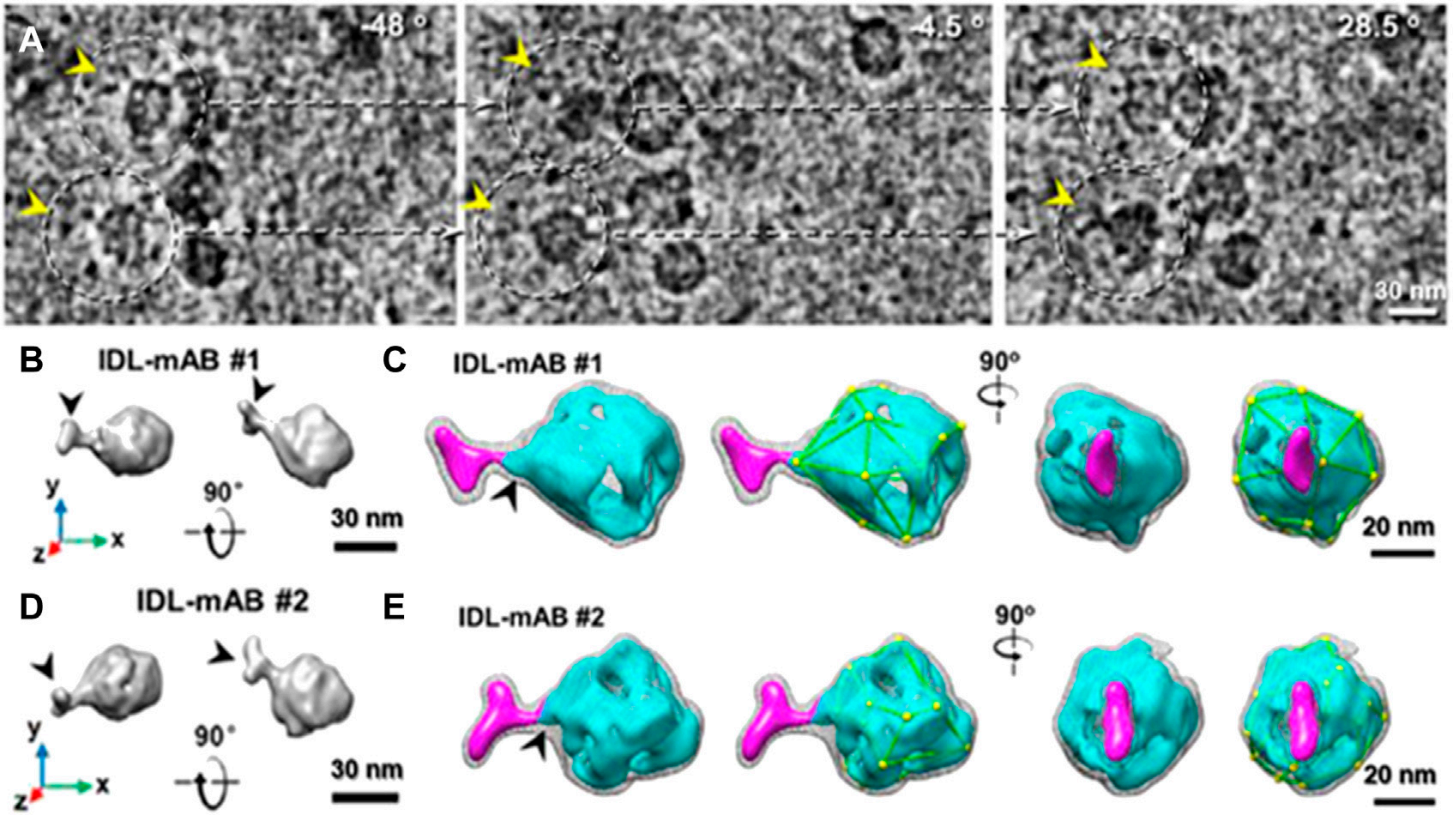
Cryo-ET 3D reconstruction of individual intermediate-density lipoprotein (IDL) particles by IPET. **(A)** Representative images of tilt series of IDL by cryo-ET. **(B,C)** Two representative views of 3D density maps of an individual IDL bound to antibody (mAB) are displayed from two orthogonal views. Maps are shown by two contour levels (high contour level in cyan surface and low contour level in gray mesh). **(D,E)** The same views for another individual IDL-mAB particle. Surface polyhedral shapes are outlined. Reproduced with permission (Lei et al., 2019).

Additionally, many computational algorithms have also been developed to enhance the image contrast (Sigworth, 2016; Fan et al., 2019a). For example, an edge-preserving smoothing–based multiscale image decomposition algorithm can detect the object against a high-noise background and enhance the object image contrast for 3D reconstruction (without averaging from different particles) of an individual small molecule (<100 kDa, CETP ∼53 kDa) (Wu et al., 2018). The missing-wedge correction software can allow us to use a small-tilt angle range for cryo-ET imaging, in which a higher dose can be used for each tilt image with the same total dose. The missing information in the missing-wedge can be restored by computational programs (Paavolainen et al., 2014; Yan et al., 2019; Moebel and Kervrann, 2020), such as the low-tilt tomography 3D reconstruction method (LoTToR) (shown in Figure 9) (Zhai et al., 2020). The application of the missing-wedge correction can also change the imaging strategy. For instance, this LoTToR allowed 3D reconstruction from a smaller tilt range with bigger tilt steps, in which the missing information can be restored by computer algorithms. As fewer number of tilt images are acquired, a higher dose can be used on each image and higher image contrast can be generated without increasing the total dose (Zhai et al., 2020). Other than the traditional algorithms, deep learning has been applied to cryo-EM image to increase the signal-to-noise ratio (Zhu et al., 2017) and to correct for the missing-wedge in 3D reconstruction (Chen et al., 2017; Ede, 2021; Liu et al., 2021) by training deep neural networks (DNNs) with cryo-ET data to improve structural interpretability in resolving lattice defects in immature HIV particles (Liu et al., 2021). The combination of the optimized data collection strategy and computational improvements, high-contrast images of single molecules can be expected in the future for high-resolution 3D reconstruction by individual-particle electron tomography (IPET) (Zhang and Ren, 2012). The resolution should be better than the current resolution, which only reveals the shape of the individual particle, such as the polyhedral shape of an individual human plasma intermediate-density lipoproteins shown in Figure 10 (Lei et al., 2019).

## Concluding remarks

By reviewing the cryo-EM/cryo-ET parameters related to the radiation damage, we wish that readers will be able to find their own optimized strategy to prevent radiation damage by maximizing the electron dose to increase their image contrast for a higher-resolution 3D reconstruction of individual molecules. For the sake of argument, we propose a strategy to maximize the tolerated dose limit for the highest-contrast imaging by selecting the following experimental parameters: the ideal cryo-EM should be a field emission gun TEM equipped with an in-column energy filter, laser phase-plate, and DED. The instrument should operate under low-voltage (e.g., ∼60–120 kV accelerating voltage) at a defocus between 0.1 and 0.4 μm, and a temperature between 20 and 60 K. The ideal specimen should be a sample imbedded in a super thin ice thickness (<∼30 nm) supported by a large hole in lacey gold films (∼5–10 μm). During the cryo-ET imaging, the angle range of ±50° in increments of 5–15° should be used for reducing the total number of acquired tilt images for higher dose exposure for each frame without increasing the total dose condition. Information in the missing-edge can be restored by computer algorithms. We expect that the optimized cryo-ET imaging will bring us to individual-molecule 3D structural studies at high-resolution, such as the tertiary structure or even the secondary structure of protein.
